# Supplementary material for: The location of an internal focus of attention differentially affects motor performance
Source: PLoS One. 2023 Nov 13;18(11):e0294246. doi: 10.1371/journal.pone.0294246 (PMC10642821; doi:10.1371/journal.pone.0294246)
Supplement: Attachment — Submitted filename: Response to reviewers.docx [file pone.0294246.s002.docx]

**Manuscript ID: PONE-D-23-04667**

**Title: The location of an internal focus of attention differentially affects motor performance**

General response: We would like to thank the Reviewers for the thoughtful comments that were provided on the previous version of this paper. Below are our replies to the specific comments provided by the reviewers. We hope that you will find our revised manuscript an improvement.

**Reviewer #1:**

**General comment:** The present study investigated the effect of different internal focusing cues in standing long jump performance. The study design was simple, which afforded clear results and interpretations. Also, the methodology was similar to the previous literature, which further strengthens the validity of the study and increases the capacity of the present results to compare and contrast with other studies. However, I have some major concerns regarding the introduction and need clarifications in the statistical analyses.

**Response:** Thank you for this comment.

**Comment 1:** Introduction (1) (minor) Line 47 and 48: the ‘motor control system’ and ‘motor behavior’ Are they used synonymously (It seems the latter has a broader meaning)? I recommend using a consistent term if not used purposefully.

**Response 1:** The term motor behavior has been replaced with motor control system.

**Comment 2:** Introduction (2) (minor) Line 54: ‘…as attention was directed at greater distances from the mover.’ I suggest adding “spatially” or “physically”: e.g., at spatially greater distances. Also, the jump distance increased as the spatial cue of the EFOA moved physically farther, not the jump distance ‘was’ increased, correct?.

**Response 2:** We included the term spatially.

**Comment 3:** Introduction (3) (minor) The second paragraph: I believe adding more contexts (regarding what parameters or factors have been investigated in SLJ) strengthens the authors’ argument and provides an even better transition to the next paragraph: (Recommendation example) I suggest providing specific factors investigated: e.g., “Since the aforementioned findings, various factors of the SLJ have been investigated, including attainability [6,7,10], skill levels [8,9], and xxxxx [REFS]”.

**Response 3:** We would like to thank the reviewer for this suggestion. We have made significant revisions to this section of our manuscript. We have added more specifics regarding the methods from the McNevin et al. (2003) and Porter et al. (2010) and Porter et al. (2012) studies to detail the distance effect on attentional focus more robustly.

**Comment 4a:** Introduction (4) (major) a. The flow of the third and fourth paragraphs is challenging to follow. I recommend a major reorganization of these paragraphs for the following reasons: a. In the first sentence of the third paragraph, the author brought up three topics: distance, relevance, and breadth of attention. This should be narrowed down.

**Response 4a:** We are confused by comment 4a. To clarify for the comment made in part “a”, the first sentence of the original third paragraph simply states that “Other research has investigated adjustments made to IFOA conditions.” We do not explicitly state the three adjustments made to investigate types of IFOA conditions: distance, relevance, and breadth.

**Comment 4b:** Introduction (4) (major) b. The authors implied, in the first sentence, that the paragraph would be about the distance of IFOA; however, the argument about the distance effect is refuted within the same paragraph, and switched to the topic of relevance without discussing interpretation, providing an approach to consolidate the issue, or providing a theoretical approach to make distinctions between the distance and relevance.

**Response 4b:** To answer part “b” of the comment the study first mentioned in the original third paragraph and now in the fifth paragraph of our revised submission, Pelleck and Passmore (2017), investigated two internal conditions compared to an external condition using a golf putting task. Of relevance to our study is the internal conditions. Pelleck and Passmore (2017) included two internal conditions: a proximal (i.e. hands and elbows) and distal (i.e. weight of their feet) focus. Their results showed that a more distal internal focus performed worse at the 5 meter distance compared to the other conditions. As they discussed in their paper, the results they found could be because of a confound in the instructions, as the proximal (i.e. hands and elbows) focus may have been interpreted as task-relevant to the golf putting task, whereas the distal (i.e. weight of their feet) may be interpreted as task-irrelevant to the golf putting task. We have added additional information to the introduction of our revised manuscript to better explain the Pelleck and Passmore (2017) study to better elucidate how task relevancy was a possible confound in their instructions designed to manipulate internal attentional focus distance.

**Comment 4c:** Introduction (4) (major) c. The criticism about the relevance was mentioned in the middle of the third paragraph, which was brought up again in the fourth paragraph after discussing different issues. This structure was very difficult to follow.

**Response 4c:** We appreciate the reviewer’s comment about the structure for paragraphs three and four, which are now paragraphs five and six in our revised draft. However, we would like to address part “c” of the comment by stating that the first sentence of paragraph five introduces the topic of “adjustments made to IFOA conditions”. Paragraph five seeks to address the modifications made to internal focus by showing previous work investigating different dimensions of attentional focus: task-relevance (Pelleck & Passmore, 2017) and breadth (Becker & Smith, 2015). In paragraph six we introduce the reader in the first sentence to “critiques” provided by other authors about the detrimental effects of an IFOA. The two paragraphs are separated since paragraph five reviews changes to IFOA conditions while paragraph six provides evidence for the claims against the detrimental effects of focusing internally. We have included the lead in “Separate from task relevancy” before mentioning the study conducted by Becker and Smith (2015) to prevent confusion of mixing different adjustments made to IFOA conditions.

**Comment 4d:** Introduction (4) (major) d. Line 77 – 80 start from: “others have proposed that…” to “the movement techniques” Although these arguments may be reasonable to bring up in the discussion, it seems irrelevant to the present manuscript. I recommend removing these sentences and focusing on the primary topic of the study (i.e., locus of internal foci).

**Response 4d:** We appreciate this suggestion. However, in our view the confound of task-relevancy is a fundamental component of choosing instructions to guide attentional focus. The Pelleck and Passmore (2017) study demonstrates that task-relevancy can possibly confound results, so we include it to accurately represent the combination of task-relevancy and distance on IFOA.

**Comment 4e:** Introduction (4) (major) e. The argument of the third and fourth paragraphs is vague: What is the critical factor affecting the previous results introduced here? The distance or relevance? What is the (potential) relationship? What do the authors desire to investigate or clarify in the present manuscript (relevance, distance, or the interaction of both)?.

**Response 4e:** To answer the first question in 4e, the critical factor in the original paragraph three, now paragraphs four and five, is to provide a background for previous work investigating changes to an IFOA. The critical factor for the original paragraph four, now paragraph six, is to show “critiques” against the findings of detrimental effects of focusing internally. To answer the second question in comment 4e, we discuss the results and interpretation of Pelleck and Passmore (2017) to show the confound of relevancy and distance when focusing internally in their study. Therefore, to answer the third question, the potential relationship between relevancy and distance a confound of moderating variables since by modifying both in the distal instruction (i.e. weight of feet) it makes it difficult to differentiate if the results observed in their study were due to the relevance or distance of the internal focus. We describe this confound in our revised paper by mentioning how the two internal focus cues given may not be an equal comparison. To answer the last question posed in part “4e” of the comment, we did not desire to investigate the interaction of relevance and distance in our study, but rather to investigate different task-relevant internal focus cues on standing long jump performance. We added a sentence to the end of the revised sixth paragraph to make that more clear for the reader.

**Comment 5:** How is the breadth of the attentional focus cues related to the purpose of the manuscript?

**Response 5:** We wrote paragraph three, which is now paragraph five, about the previous work that has investigated adjustments made to IFOA conditions. Specifically, we included the work from Becker and Smith (2015) since they adjusted the breadth of internal focus. The previous work on breadth relates to the purpose of the manuscript since it still leaves a gap in the research related to the purpose of our paper. Moreover, paragraph five reviews previous work done investigating other modifications to an IFOA, particularly regarding how it is related to the standing long jump.

**Comment 6a:** (major) Before the authors provided their hypotheses, it didn’t seem that the problem of the previous studies (distance vs. relevance) was not consolidated: a. What is the rationale for choosing these different body parts specific to the relevance of the cues and specific to the distance of the cues?

**Response 6a:** We appreciate the question posed by the reviewer. We chose body parts that are task-relevant to the standing long jump. Specifically, we selected the action of body movements which are commonly referenced when instructing the standing long jump. We have made this more clear in the revised manuscript by stating that we “sought to specifically investigate how jump distance was impacted by various forms of an IFOA relative to jumps completed in a control condition.”

**Comment 6b:** (major) b. What is the rationale for hypothesizing all cues, regardless of the distance and relevance, are equally poorer than an EFOA? For instance, the authors discussed the results regarding the breadth of the focus of attention, which was not different by its factor. However, the authors mentioned that the relevance OR distance did affect the results. Then, the hypothesis is inconsistent with the previous study (Pelleck & Passmore), which is fine, but why?

**Response 6b:** We appreciate the reviewer’s question about the rationale for our hypotheses. Since previous work has shown that internal cues have a detrimental effect on standing long jump performance, we predicted that would be true regardless of the location for an IFOA. This prediction was the product of the limited work examining the adoption of an internal focus of attention when executing the standing long jump. Additionally, we would like to clarify the results and interpretation of the Pelleck and Passmore (2017) paper. Specifically, their investigation included the modification of both relevance and distance of internal focus rather than the two being investigated independently. Therefore, we do not feel it is valid to conclude their results would directly frame our hypotheses since we did not want to confound our IFOA conditions with task-irrelevance. To address the point about inconsistency, our hypotheses does not seem to be inconsistent with their findings as Pelleck and Passmore (2017) considering they did not include a control condition nor did they separate the confound of relevancy for their internal focus conditions.

**Comment 6c:** (major) What is the ‘relevance’ of each IFOA cue? Any support from biomechanics, motor control, or strength & conditioning literature?

**Response 6c:** We selected instructions which reference gross body movements which are commonly used when instructing the standing long jump.

**Comment 7:** (minor) Participants: Although I am not a strong proponent of blindly relying on the power analysis (because it is more complicated than how they have been used), providing justifications for the sample size is important.?

**Response 7:** The sample size used in our study is consistent with previous research examining the focus of attention effect using a jumping task. We have made this clear in the revised manuscript and added a citation.

**Comment 8:** (minor) Participants: Please justify why only males were recruited (a sentence or two would be sufficient).

**Response 8:** Only males were included in the sample to eliminate sex based jumping difference. This method is common in previous focus of attention studies which uses power based tasks. There is no evidence that males and females respond differently to the focus of attention effect. However, there is evidence that males jump further than females. To minimize the inherent variability of the sample, only males were recruited.

**Comment 9:** (minor) Participants: How were they recruited? What are the characteristics of the participants? (who can the cohort be generalized?)

**Response 9:** Participants were recruited from a variety of courses at a large university in the United States of America. We have added the following sentence to the revised manuscript to better describe the sample “None of the participants received previous training in the SLJ. Additionally, none of the volunteers were current or former collegiate or professional athletes.”

**Comment 10:** (minor) Apparatus and task: Was the order completely randomized or pseudo-randomized? That is, was there any order effect at all?

**Response 10:** We changed the term counterbalanced to randomized. No order effects were observed.

**Comment 11:** (major) How was Type 1 error controlled?

**Response 11:** We appreciate the question from the reviewer. A Mauchly's test of sphericity was conducted and we observed that no violation occurred (*p* > .05). We have mentioned Mauchly’s sphericity test in statistical analysis section of the revised paper. We have also included the results of Mauchly’s test of sphericity in the first sentence of the results section.

**Comment 12(1):** (major) Was there any missing data?

**Response 12(1):** We appreciate the questions from the reviewer. There was no data missing in our study.

**Comment 12(2):** (major) How did you validate the assumptions?

**Response 12(2):** We do not understand the question about validating assumptions.

**Comment 12(3):** (major) Were there any outliers?

**Response 12(3):** There were two outliers in the hip condition. However, removal of the outliers led to no statistical differences in the analyses. Therefore, we decided to keep all of the data for the analysis.

**Comment 12(4):** (major) How were they [outliers] decided to be treated before data analysis?

**Response 12(4):** Outlier removal was not used in our analysis; therefore, all the collected data were used in the analysis.

**Comment 13:** (major) How can the degree of freedom be 28 where the sample size was 26?

**Response 13:** We appreciate question about the degrees of freedom. There was a typo for the sample size of 26. The actual sample size was 29 participants. We have made the change to 29 participants in the participants section of the paper.

**Comment 14:** Could you provide a table of M and SD?

**Response 14:** If we were to add a table of mean and standard deviation for each group then it would be providing redundant information with the data represented in the Figure 1 bar graph. It is for that reason we did not include both a table and figure of the same data.

**Comment 15:** Could you replace the figure with an alternative method? E.g., superimposing raw data over the current bar graph, adding a violin plot next to the current graph, or superimposing the mean and SE points over a box plot (or simply, a box plot). Bar graphs do not show the distribution of the data, so it’s not reader-friendly to demonstrate the validity of the statistical choices.

**Response 15:** We thank the reviewer for the question about an alternative figure. We did try to create a variety of figures to present the data, which can be observed below this response. In the left column we have the original graph given in the first submission and on the right column includes the alternatives. In all the alternative graphs, we can see that including the entire distribution of data set, whether as a violin, jitter, or jitter with a violin, occludes the reader from observing the differences between groups since the y-axis has to be elongated to include the distribution of data. Moreover, since the study was a within-participant design we should expect to see similarities in the standard error (also we did not violate Mauchly’s test of sphericity) which can be observed more easily in the original graph. All alternative graphs shrink the standard error bars making it hard to see them. Additionally, previous work on standing long jump has used bar graphs to illustrate the data. We would not want to confuse readers with different presentations of the data and keep in line with previous work (see Porter et al., 2010; Porter et al., 2012, Coker 2016, Westphal & Porter, 2013; Becker & Smith 2015; Duscharme et al. 2016). As a result, we feel the clearest representation of our data exist in a bar graph with standard error bars.

| Original | Alternative Bar with Jitter Plot |
| --- | --- |
|  |  |
| Orignal | Alternative Violin Plot |
|  |  |
| Original | Alternative Violin Plot with Jitter Plot |
|  |  |

**Reviewer #2**

The article entitled “The location of internal focus of attention differentially affects motor performance” presents a study in a long line of research on the effects of attentional focus on performance and learning. Typically, research on attentional focus has centered on the benefits of an external focus compared to a control or internal focus of attention. Consistently, an internal focus of attention has found to be less beneficial than an external focus. The present study sought to investigate various locations of internal focus compared to a control condition. The findings indicate that performance during the internal focus condition was consistently worse on a standing long jump compared to a control condition. Four different locations of internal focus were examined, and a significant difference emerged between performance focus on the knees vs arms.

While the study is generally well written, there are a number of methodological issues that prevent one from drawing conclusions about the results. I will present my general concerns below, followed by specific comments.

**GENERAL COMMENTS**

**Comment 1:** The authors test a variety of locations of internal focus, but what was the reasoning behind the locations selected? It should be the case that the internal focus locations examined should cover the range of possible variations of internal focus characteristics (e.g., proximal, distal, relevant, irrelevant, etc). The authors should justify their selected choices. For example, if I selected a collections of internal focus locations (e.g,. pointer finger, pinky finger, thumb), I could easily show no difference between internal focus conditions, and potentially wrongly conclude that all internal focus locations are equally detrimental.

**Response 1:** We appreciate the comment from the reviewer. To answer the question, we state that the purpose of this study was to investigate if a variety of internal focused locations would lead to determents to standing long jump performance compared to a control condition. To best address the purpose of the experiment, we manipulated the internal focus locations to a variety of body parts that are relevant to the standing jump (i.e. toe, knee, hip, arms). We did not intend to exhaustively test all possible variations and combinations across a number of different internal focus dimensions (i.e. location, breadth, relevance, distance, location and breadth, location and relevance, location and distance, breadth and relevance, breadth and distance, relevance and distance, location and breadth and relevance, etc.). Examining all dimensions of internal focus manipulations is beyond the scope of our study. The critique posed by the reviewer about choosing the following internal focus locations as “(e.g. pointer finger, pinky finger, thumb)” is the same critique posed by Herrebrøden (2022) and Montero (2019) as to the relevance of the internal or external cues given. We address the confound of task-relevance and distance by including the Pelleck and Passmore (2017) study, as we illustrate that there manipulation of distance for the internal focus conditions could be a confound for a separate dimensions of task-relevance. Furthermore, we include the critiques posed by Herrebrøden (2022) and Montero (2019) to address the appropriateness and relevant comparison of instructions for a given motor skill, like the standing long jump. In short, we desired to only investigate internal cues which were task relevant to the performance of the standing long jump. Additionally, we have added a clarification sentence at the bottom of paragraph six in our introduction to show that we choose cues that would not confound task-relevancy with task-irrelevancy since we are just manipulating location. We feel all internal focus locations selected in our study are relevant to performing the standing long jump.

**Comment 2:** The authors claim that an internal focus is detrimental. However, are the authors actually able to claim it is “detrimental”? Does an internal focus actively hurt performance, or does an external focus improve performance relative to internal focus which has no effect on performance at all? For example, if the participants adopted an internal focus, would that result in worse performance, than if they focused on the color blue (assuming that focusing on a color would have zero impact on performance). This leads me to another concern with the methodology. One can’t assume that the control condition acts as a baseline because we don’t know what the participants were focusing on. The lack of manipulation check hampers the ability to determine how to interpret control condition performance. For example, research exists that suggests under control conditions, participants tend to adopt an internal focus of condition. If this happened in the present study, then you essentially have 5 different internal focus conditions, and thus we see differences across internal focus locations in contradiction to the hypothesis of the study?

**Response 2:** The results of our study allow us to conclude that instructing an internal focus resulted in performance decrements. This idea is explained by the constrained action hypothesis (see Chua et al., 2021 for a review). Other work looking into a variety of motor performance measures have observed performance decrements when focusing internally like in reaction time for both skilled and unskilled sprinters (Ille et al., 2013), reaction time in a balancing task (Wulf et al., 2001), reaction time in a probe-reaction time task compared to control condition (Adams et al., 2014), decreased golf putting performance (Wulf et al., 1999; Wulf & Su, 2007; Kearney 2015; Bell & Hardy, 2009), decreased balancing performance (Wulf et al, 1999; Chiviacowsky et al., 2010, Diekfuss et al., 2018), decreased standing long jump performance (Porter et al., 2010; Ducharme et al., 2016), decreased sprinting performance (Porter & Sims, 2013, Porter et al., 2015), decreased dart throw performance (Hitchcock & Sherwood, 2018; Lohse et al., 2010) to name a few. It has been observed in the work of previous authors, supporting the predictions made by the constrained action hypothesis (Wulf et al., 2001) that an internal focus is detrimental to motor performance.

We appreciate the concern from the reviewer about the question posed comparing a cue to attentional focus on the color blue compared to an internal focus. There are multitude of considerations that should take place when investigating that question. For instance, where is the color “blue” located? Our answer to the original question posed would also depend on the location of the color. If the color is located on a cone placed on the standing long jump mat versus the color placed on the participant’s knee may differentially affect standing long jump performance. However interesting that question may be, we do not believe that idea is relevant to the work present in our paper. Our paper investigated a variety of internal focus locations, not a possible task-relevant or task irrelevant cue like the color blue presented in this comment.

We understand the concern raised by the reviewer regarding a manipulation check. However, this is an inherent limitation in all human based research that utilize instructions. Even with a manipulation check, it is impossible to ensure that participants complied with the instructions. If a participant reports that they did exactly what they were instructed to do, it does not guarantee that they actually did follow the instructions. It’s impossible to know. We tried to reasonably ensure compliance through the experimental design. Meaning that all conditions were treated equally except for the prescribed instructions. So if the groups performed differently, it strongly suggest it was a result of the prescribed instructions. Furthermore, such a finding suggest that participants complied with the prescribed instructions considering they are performing differently. If participants were not following the instructions, then it would be expected that all groups would perform equally, perhaps even identically. Which is not what the results of the present study demonstrated. Considering this is an inherent limitation for all research that utilize instructions, we do not see this as a unique limitation of our study.

**Comment 3:** It appears that the familywise error rate was not taken into account when performing the multiple post hoc comparisons. If one were to implement a Bonferroni correction, then the difference between these two internal conditions would no longer significant. .05/5 = .01 (required significance level).?

**Response 3**: We appreciate the comment made about the Bonferroni correction. It is possible that there may not be a statistically significant difference between the internal knee and internal arm conditions if were to perform a familywise Bonferroni post-hoc analysis. We agree that performing a Bonferroni post-hoc analysis would yield a divided alpha level (α = .01) among groupwise comparisons. However, doing this procedure could lead to an overly conservative approach thereby allowing for the possibility of a type II error. Our initial alpha level (α = .05) is still extensively used by the majority of research areas using the frequentist statistical approach across a variety of fields of study. Therefore, we decided to keep the same alpha level for pairwise comparisons with the simple effects analysis.

**SPECIFIC COMMENTS**

**Comment 4:** Line 46. This is a limited explanation of constrained action hypothesis, and could use a more accurate and detailed explanation of this. For example, explain what is meant when you say system operates more autonomously. Saying that behavior is disrupted doesn’t explain how internal focus hurts performance. This is vague. In what way is it disrupted?

**Response 4:** Thank you for the feedback. We have made significant revisions to this section of the manuscript. We provided a more robust explanation of the constrained action hypothesis and have also cited additional research to demonstrate previous findings on this topic. Specifically, we mentioned studies looking into bio-behavioral support as observed in studies using electromyography (EMG), movement production, and central nervous system activity. These additions can be seen following the mention of the constrained action hypothesis in paragraph two of the introduction.

**Comment 5:** Line 51. You need to better explain what you mean by distance of an EFOA. You need to clarify that you are referring to distal and proximal relationship to the body. A reader with no background in this topic wouldn’t understand what you mean by distance (e.g., distance of what?). You reference this later in the paper, but could be explained earlier when you initially start talking about distance of EFOA..

**Response 5:** This is a fair point. We have elaborated and explained the McNevin et al. (2003) and Porter et al. studies to better clarify the distance effect on EFOA. In the case of McNevin et al. (2003), we added the locations on the stabilometer platform for near versus far EFOA. Additionally, we included the instructions stated by Porter et al. (2012) to elucidate the difference between near and far EFOA as it pertains to the standing long jump. These changes can be found in paragraphs three and four of the introduction.

**Comment 6:** Line 54. Provide examples of different types of external distance instructions used in this study, in case the reader hasn’t read this article.

**Response 6:** We have added examples from the Porter et al. (2012) study for instructions comparing the distance effect for EFOA. Those changes can be found in paragraph four of the introduction.

**Comment 7:** Participants: Why only male participants? Were they novice at the SLJ?

**Response 7:** Only males were included in the sample to eliminate sex based jumping difference. This method is common in previous focus of attention studies which uses power based tasks. There is no evidence that males and females respond differently to the focus of attention effect. However, there is evidence that males jump farther than females. To minimize the inherent variability of the sample, only males were recruited. We have added the following sentence to the revised manuscript to better describe the sample “None of the participants received previous training in the SLJ. Additionally, none of the volunteers were current or former collegiate or professional athletes.”

**Comment 8:** Line 121: Please state exactly what the participants were told for the internal focus conditions. What was the exact phrase used?

**Response 8:** The exact instructions have been added to the revised manuscript in the Procedures section.

**Comment 9:** Line 123: Was the control condition always performed first? No mention of the order of conditions.

**Response 9:** The control condition was not always performed first, it was randomized with the four internal conditions. In the last sentence, of the apparatus and task section of the paper we note how the conditions were ordered. We replaced the term counterbalanced with randomized to more accurately reflect the methods used in this study.

**Comment 10:** Results: Looking at Fig 1, while there are significant differences, it appears that the difference in jump length is only a few centimeters. Is this meaningful?

**Response 10:** We feel that significantly changing horizontal jump distance with only a couple of jumps within a single practice session is meaningful. The results of our study show statistically significant differences between control and all IFOA conditions. This suggests that the findings in our study are highly unlikely to be the result of chance or a type 1 error. Moreover, the effect sizes from our study in terms of Cohen’s *d* show a medium effect size of nearly a half a standard deviation (arm to knee comparison) to three quarters of a standard deviation (control to knee comparison). These effect sizes show a meaningful difference between experimental conditions.

**Comment 11:** Line 153: “movers” should be “mover’s”.

**Response 11:** The change was made.

**Comment 12:** Line 157: Is it determinantal, or does it just not provide anything helpful?

**Response 12:** We do not understand how the determinant of our data is relevant to the comment, so we are assuming that the reviewer meant “detrimental”. Standing long jump performance was shorter in all internal focus conditions compared to the control condition. It is in our view that a decrease in performance would be considered detrimental. If an internal focus of attention did not provide anything helpful then the results of our study would have shown equal or equivalent standing long jump performances across all experimental conditions. We did not observe such a finding in our data.

**Comment 13:** Line 172: Since you don’t know what people will focus on in the control condition, how can you predict that performance will be different? What if people in the control condition focused on their body movement? Research shows that there is a tendency to adopt an internal focus on sports skills.

**Response 13:** We understand the concern raised by the reviewer. To be honest, we don’t know what participants focused on in any of the conditions. This is an inherent limitation in all human based research that utilize instructions. Even with a manipulation check, it is impossible to ensure that participants complied with the instructions. If a participant reports that they did exactly what they were instructed to do, it does not guarantee that they actually did follow the instructions. It’s impossible to know. We tried to reasonably ensure compliance through the experimental design. Meaning that all conditions were treated equally except for the prescribed instructions. So if the groups performed differently, it strongly suggest it was a result of the prescribed instructions. Furthermore, such a finding suggest that participants complied with the prescribed instructions considering they are performing differently. If participants were not following the instructions, then it would be expected that all groups would perform equally, perhaps even identically. Which is not what the results of the present study demonstrated. Considering this is an inherent limitation for all research that utilize instructions, we do not see this as a unique limitation of our study.

**Comment 14:** Line 178: The difference between these internal conditions appeared to be very small (approximately 2cm). It would be more helpful to provide a relative change in jump length (i.e., 1% increase in jump length). Need to provide the average length of the jumps, then the reader can determine how meaningful 2cm actually is.

**Response 14:** First, we would like to clarify that all participants performed two standing long jumps per condition and the average of those two jumps was used in the analysis. The advice about percentage change is something to consider, however, we did not conduct a baseline measurement before we began the experimental conditions. Without a baseline measurement as a reference point it would be difficult to make an assumption of what would be a percentage distance increase or decrease would be per condition. Moreover, if we chose to use one of the experimental conditions as a baseline we would lose the reference to itself, because it would be equal to 0% difference. It is for those reasons that we appreciate the comment, but we choose to maintain the distance as stated in the manuscript. Our reporting of group performance is also consistent with previous research on this topic.

**Comment 15:** Line 186: Can automaticity be anything other than self-organized?

**Response 15:** As it pertains to the present paper, automaticity in the motor control system is a self-organized non-conscious process. We did not investigate what components or systems are self-organized as that was not the purpose of this study.

**Comment 16** Line 190: “significantly” only with respect to statistics. I’m not convinced this small difference is meaningful.

**Response 16:** Our results show statistical significance and medium effect sizes in our follow-up simple effects analysis. Therefore the results of our findings, not only are statistically significant, but also the effect sizes between conditions are meaningful. Future readers can determine if our findings were meaningful in their eyes.

**Comment 17:** Discussion: Given the possible reasons for why various internal focus conditions might lead to the observed differences between the legs and arms, why did the researchers predict no differences between the various internal focus conditions. Clearly there are logical reasons why difference might have existed.

**Response 17:** We appreciate the comment. We made this prediction because previous work looking at similarly skilled adults performing the standing long jump had shown detrimental performance in internal conditions. Additionally, following the predictions made by the constrained action hypothesis we hypothesized that internal focus location is not a relevant mediator for the disruption of the motor control system. There can be many logical reasons that are not as well grounded in literature as the predictions made by the constrained action hypothesis, and we offer those alternatives in the discussion regarding electromyography in paragraph three, distance effect in paragraph four, and momentum in paragraph five. To our knowledge, none of the explanations we offered have been rigorously tested so we did not make predictions for differences between IFOA conditions before we conducted this study, as we did not feel such a prediction was justified based on current research.

**Comment 18:** Line 215: I’m not sure how arms are considered more distal and legs. From what point are you measuring from?

**Response 18:** We appreciate the question about the measurement regarding the distance effect. We state in paragraph four that origin point is the “center of gravity” which found in paragraph five. Additionally, we did not have a “leg” condition, but we had a “hip”, “knee”, and “toe” condition which represent different reference point on the “leg”.

**Reviewer #3**

**GENERAL COMMENTS**

**Comment 1:** A within-participants experimental design was used in this study to investigate the differential effects on horizontal jump distance of adopting four types of internal attentional focus and no attentional focus during standing long jump. It was found that participants jumped farther in the control condition as compared to any of the four internal focus conditions. Participants also had better jump performance when adopting an internal focus on the arm than the knee. There are several major concerns regarding literature review accuracy, methodological rigor, figure accuracy, and basis of discussion points. Several minor recommendations/suggestions were provided in the hope that the authors would find them helpful for improving the overall quality of the manuscript.

**MAJOR COMMENTS - INTRODUCTION**

**Comment 2:** Pg. 4 Lines 72−73: Coker (2018), Neumann and Piercy (2013), Pelleck and Passmore (2017), Schϋcker, Fleddermann, de Lussanet, Elischer, Bohmer, and Zentgraf (2016), Schϋcker, Hagemann, Strauss, and Volker (2009), Schϋcker, Schmeing, and Hagemann (2016), Hill, Schϋcker, Hagemann, and Strauβ (2017), and Oki, Kokubu, and Nagagomi (2018) also compared various types of internal focus cues with external focus cues.

**Response 2:** We appreciate mentioning other references comparing internal focus cues. After looking through all the provided references we decided to include the papers from Coker (2018) and Oki, Kokubu, and Nagagomi (2018) as they provide good insight for the purpose of the present manuscript. We have included those two papers in paragraph five of our revised introduction. However, after reading the other references we do not think they are appropriate for the purpose of our paper as they do not compare multiple different internal focus conditions nor different variations of internal focus.

**Comment 3:** Pg. 4 Lines 82−83: See above comment regarding other studies that examined various types of internal focus.

**Response 3:** We appreciate this comment. Please see our response to comment two above.

**MAJOR COMMENTS - METHODS**

**Comment 4:** Pg. 5 Line 98: Was this sample size recruited based on a statistical power analysis?

**Response 4:** The sample size used in our study is consistent with previous research examining the focus of attention effect using a jumping task. We have made this clear in the revised manuscript and added a citation.

**Comment 5:** Pg. 5 Line 105: As a general guide, only aspects of the task environment that are known to significantly influence motor performance or would be manipulated as required by the design of the intervention need to be described for replication purposes. And for such purposes, adequate details should be provided for reference by other researchers. Does climate control serve these purposes?

**Response 5:** Yes, a climate controlled laboratory helps to describe the environment that was used in this study. This was not an outdoor or field based standing long jump study. As a result, weather or ambient temperature did not fluctuate between participants. Both of which are features of the task environment, if not controlled, may impact motor performance.

**Comment 6:** Pg. 5 Line 109: Specify the thickness of this line.

**Response 6:** The thickness of each measurement line was 0.318 centimeters. We have included width of each line in the apparatus and task section of the manuscript.

**Comment 7:** Pg. 5 Lines 112−113: It is mathematically impossible to counterbalance 120 ways of arranging five conditions across 26 participants.

**Response 7:** We changed the term counterbalanced to randomized to accurately reflect the methods used in this experiment.

**Comment 8:** Pg. 6 Lines 119−120: Provide examples of how these IFOA cues were used in the instructions given to participants.

**Response 8:** The exact instructions have been added to the revised manuscript it the Procedures section.

**Comment 9:** Pg. 6 Line 131: Were the data checked for normality of distribution to ensure the valid use of ANOVA?

**Response 9:** We appreciate this comment. We conducted a Shapiro-Wilk test for each experimental condition and found that only the Hip condition (*p* = 0.48) violated the normality assumption. However, there was not a severe departure from normality. Given these findings we still used a repeated measures ANOVA. We added a statement in the statistical analysis section to mention the Shapiro-Wilk test.

**Comment 10a:** Pg. 16 Figure 1: Why are all the error bars of equal length?

**Response 10a:** We appreciate this question. The error bars are nearly all equal length because of the within subjects design of the experiment. Since we had the same participants for all conditions we expected to observe equal variances across the conditions. This was confirmed when we did not violate Mauchly’s test of sphericity (*p* >.05) and therefore we can therefore assume equal variances. We added a mention of Mauchly’s test of sphericity in both the Statistical Analysis and Results sections of the paper.

**Comment 10b:** Pg. 16 Figure 1: What are the SE values for these five jump distances?

**Response 10b:** The standard errors for the experimental conditions are as follows: toe (*SE* = 3.89), hip (*SE* = 4.11), knee (*SE* = 4.22), arm (*SE* = 4.13), and control (*SE* = 4.62).

**MAJOR COMMENTS - DISCUSSION**

**Comment 11:** Pg. 8 Line 171: The terms within the parentheses are more appropriately described as cues rather than instructions. The use of “in addition” here suggests that internal focus instructions were added to neutral instruction given in the control condition.

**Response 11:** We have changed this to “cues.”

**Comment 12:** Pg. 9 Line 189: What does the “generalizability of IFOA” mean?

**Response 12:** Generalizability of IFOA here means that all internal foci, regardless of location, can disrupt the automatic processes of the motor control system, thereby resulting in performance decrements.

**Comment 13:** Pg. 10 Line 215−218: Would the hips not be a more proximal IFOA cue than the knees? This “center-of-gravity” hypothesis would predict that an internal focus on the arms would also allow participants to jump farther than an internal focus on the hips. This difference, however, was found to be non-significant in this study.

**Response 13:** We appreciate the comment and the question posed by the reviewer. We added a section mentioning that we did not observe significant differences between the hip and knee conditions. We explain that it may be due to the relatively short distance between the hips and knees that may not uncover statistically significant differences in standing long jump performance. Moreover, we add the distance between arm and center of gravity may have been enough to see the internal distance effect on standing long jump performance. Specifically, we hypothesize that there could be a necessary distance threshold that is required to observe this internal distance effect, which was seen in the arm condition and not in the other IFOA conditions.

**Comment 14:** What are the limitations of this study?

**Response 14:** The biggest limitation is that we only tested college-aged males in this study, none of which were highly skilled. In order to test the generalization of IFOA locations more participants from diverse populations will be required.

**Comment 15:** How could the findings of this study be applied?

**Response 15:** We appreciate the question from the reviewer. We conclude our paper with a practical application indicating that jumpers should direct their attention externally rather than internally. Moreover, we propose that jumpers should not be instructed to direct their attention internally.

**MINOR COMMENTS**

**Comment 16:** Pg. 2 Line 44: Does this emergence refer to the empirical evidence on these differences?

**Response 16:** We have removed the word “emerging” since the sentence is referring to previous work that has already been published or “emerged”.

**Comment 17:** Pg. 3 Lines 64−65: Different types of internal focus are not meant to be the same, but may share similar aspects. Consider replacing “equal” with “equivalent”, or its synonym, to reflect this conceptual difference.

**Response 17:** This change was made.

**Comment 18:** Pg. 3 Line 68: Use the past tense of “lead” and add “with” after “line”.

**Response 18:** The change was made.

**Comment 19:** Pg. 4 Lines 84−85: The conditions per se could not produce the performance, but it is the use of these conditions that could lead to differences in participants’ performance.

**Response 19:** We have changed the sentence to reflect the observation of performance decrements in the internal focus conditions compared to the control condition.

**Comment 20:** Pg. 4 Line 86: Consider adding “conditions” after “IFOA”.

**Response 20:** We have made the change.

**Comment 21:** Pg. 7 Lines 140 and 147−148, and Pg. 9 Lines 180−181: The conditions per se could not make the jump, but the jumps were made by participants under these conditions.

**Response 21:** We have changed the sentence to show that standing long jump performance was observed in the control condition compared to the other conditions.

**Comment 22:** Pg. 7 Line 142: Standardize the number of decimal places used to report p values.

**Response 22:** The change was made.

**Comment 23:** Pg. 8 Line 166: The use of “argue” is usually associated with the presentation of two opposing views.

**Response 23:** We have changed the word “argued” to “questioned” in the revised manuscript.

**Comment 24:** Pg. 9 Line 175: “led”.

**Response 24:** We have made the change.

**Comment 25:** Pg. 10 Line 205: Add “to” after “due”.

**Response 25:** We have made the change.
