# Supplementary material for: The location of an internal focus of attention differentially affects motor performance
Source: PLoS One. 2023 Nov 13;18(11):e0294246. doi: 10.1371/journal.pone.0294246 (PMC10642821; doi:10.1371/journal.pone.0294246)
Supplement: Attachment — Submitted filename: Response to reviewers.docx [file pone.0294246.s003.docx]

**Manuscript ID: PONE-D-23-04667**

**Title: The location of an internal focus of attention differentially affects motor performance**

General response: We would like to thank the Reviewers for their contributions and thoughtful comments to this updated version of the manuscript. Below are our replies to the specific comments provided by each of the reviewers. We hope that you will find our revised manuscript an improvement.

**Reviewer #1:**

**General comment:** The revised manuscript has been immensely improved. I agree with most changes. However, there was a slight misunderstanding in response to my comments (Point 6); some of the comments were responded to but without any revisions on the manuscript (point 3, 6, 7, and 8); and one response was unclear (point 5). Most importantly, I cannot agree with the responses to Comments 7, 14, and 15 from the revision (point 2 and 10). Thus, I recommend minor revisions by either clarifying these points and/or providing scientific and rational justifications that go against the current guidelines of science.

**Response:** We appreciate the complements. We have responded to each specific comment below.

**Comment 1:** Response to Comment 7: Just because the previous literature used the same sample size can raise tremendous counterarguments, especially from those who are familiar with methodology/statistics. The lack of justification can lead to statistically unethical procedures, e.g., (a) collecting data until the investigator found significance; (b) stopping data collection when significance was found without justification; (c) simply unreliable results. That is, even if p = 0.05, if the power is low, the conclusion cannot be confidently made about the rejection of the null hypothesis; and (d) none-informative studies, i.e., a small effect size that may not have practical meaningfulness. For example, Porter et al. (2010) collected N = 120, which is one of the largest samples ever collected in the motor learning area. The effect size returned negligible (i.e., d). Other studies collected much less sample size (partially due to the study design), but none of the cited articles in the manuscript (Becker & Smith, 2015; Cocker, 2016, 2018; Ducharme et al., 2016; Hubert & Williams, 2017; King & Porter, 2021; Mirmiran et al., 2019; Nagano et al., 2020; Porter et al., 2010, 2012, 2013, Westphal & Porter, 2012) provided a written justification or reported a priori power analysis. Unfortunately, the research in motor learning is clearly biased and difficult to replicate (McKay et al., 2022a) because of some of the reasons mentioned above. These poor statistical ethics has led to conclusions that many previous research topics in motor learning/control (i.e., autonomy, expectancy, reduced feedback) are highly biased and their effects are negligible (McKay et al., 2022b, 2023), including external/internal focus (Mckay et al., under review).

Given that, just that statement (using the sample size from previous literature with no (report of) prior power analysis) can lead to rejection nowadays. Therefore, for your defense, I strongly encourage the authors to add a limitation that the authors failed to perform a power analysis that can potentially lead to a lack of statistical rigor, and future studies should ensure analysis with high statistical power.
Reference
McKay et al. (under review): <https://sportrxiv.org/index.php/server/preprint/view/304>
McKay et al. (2022a): <https://journals.humankinetics.com/view/journals/jmld/11/1/article-p15.xml>
McKay et al. (2022b): <https://www.sciencedirect.com/science/article/abs/pii/S1469029222000334>
McKay et al. (2023): <https://psyarxiv.com/3nhtc/>

**Response 1:** We conducted a post-hoc power analysis on our data and found that we reached adequate power for the sample (1-β > .99). We have added a reference to the software package used in the statistical analysis section and the results of the post-hoc power analysis to the results section of the paper. Additionally, we added limitations to the last paragraph of the discussion about the participant pool selected for the study design. We do mention that future work should investigate if the results of this paper are generalizable to more diverse populations.

**Comment 2:** Response to Comment 8: I was looking for the authors’ response, “Only males were included in the sample to eliminate sex based jumping difference.” I believe this is a sufficient reason to justify the choice of the particular sex. As sex difference and sex inclusion are extremely valued in the current scientific field, please add that response to the manuscript for your defense.

**Response 2:** We have added a statement in the participants section for the rationale for including only males in this study.

**Comment 3:** Response to Comment 10: The response does not make sense. If it was truly randomized of 5 potential orders, that’s 120 possible orders? How was the order effect tested? If it was counterbalanced, the order effect could be tested. However, the authors mentioned that ‘no order effects were observed,’ and the term was changed from counterbalanced to randomized. If it was truly randomized, there was no way to test or observe the order effect as it has too many variations, so we just need to hope there would be no bias based on the probability theory. Please explain this in detail.

**Response 3:** We feel the language in the revised manuscript is sufficient. The method we used in the study to randomize order is incredibly common in the human sciences. Based on a suggestion from reviewer 3, we have added a table in the apparatus and task section that includes the count and percentage of participants that had each condition first or last.

**Comment 4:** Response to Comment 11: Type I error is inflation of p-values due to multiple comparisons. I see that the authors performed post hoc tests, not a priori contrasts. (a) How was the inflation of error controlled for the multiple comparisons? (b) Are the p-values of the post hoc tests raw values or values after controlling for the type I error with some methods (e.g., Bonferroni)?

**Response 4:** We appreciate the comment about post-hoc corrections from the reviewer. In our analysis of the data we followed statistical procedures that have been used in previous work using the standing long jump task (Becker, Fairbrother, & Couvillion, 2020; Porter, Anton, & Wu, 2012; Wu, Porter, & Brown, 2012). Even though we state that we use simple effects paired tests for our post-hoc analysis it is the same procedure as done with post-hoc least significant difference testing stated in the citations provided above.

**Comment 5:** Responses to Comment 12(1,3,4): About outliers. Thank you for the clarification. Please add your responses to the analysis section (i.e., how many outliers and missing data were there; how outliers were treated and the rationale for removing or not removing outliers). As this information affects the future synthesis (if you do not describe missing or outliers, and there was a difference in the degrees of freedom, readers would not be able to tell if it is a typo or due to some data processing), it’s important that the authors add them to the manuscript.

**Response 5:** This information has been added to the revised manuscript. Specifically, it is included following the results from the Shapiro-Wilk test.

**Comment 6:** Response to Comments 14 & 15: In response to my recommendations to (a) present M, SD, and (b)replace bar graphs with other forms of figure, the authors responded (a) that would be redundant to figures and (b) bar graphs are consistent with previous literature, and other forms of figures are not more informative than the bar graph.

I cannot agree with both comments. First, the scientific literature should contribute to the development of science. This includes contributions to the future synthesis of knowledge (i.e., meta-analysis) and keeping up with the most recent guidelines based on accumulated knowledge from various areas of science.

For (a), without M, df, and SD (and ideally 95% CI or SE), the literature cannot be used for meta-analysis. This does not only lead to stagnant of scientific development but is also disadvantageous for the authors for not being included in the future synthesis of knowledge. Thus, figures and the actual numbers are not redundant. I see that the authors added M and SE. However, it would be more researcher-friendly if you could add M, SD, and 95% CI to the result section, especially as the authors used SPSS in the analysis. Moreover, the authors added M and SE selectively (I assume only for significant findings). This tradition has been harshly criticized as it produces bias. Thus, either adding all results in the text or simply adding a table would be a better option.

For (b), I clearly see the benefits of the authors’ alternative plots. First, readers can see the spread of the data. Second, readers can see the distribution of the data. Thus, readers can assess whether the results are reliable (i.e., statistical assumptions are met). Third, readers can also see outliers. The debate about how to deal with outliers has not been resolved, and that is why it is important to show and describe the rationale for removing or not removing outliers. Fourth, yes, the alternative graphs may be more visually difficult to see differences; however, the bar graph can lead to exaggerated interpretation as the y-axis range is only 70 – 84 on the left column figures. I applaud the authors for adding asterisks to indicate the source of statistical significance.

There has been a strong shift of moving away from “bad” scientific traditions (not because the past researchers were bad but because there have been improvements), and one of them is the use of bar graphs. Therefore, I respect the authors for trying to be consistent (which is great, and others don’t do that!), but maintaining bad traditions is a different topic. There are numerous criticisms you could find, but for reference, please see Weissgerber et al. (2019).

I do not have a strong position on statistical tests for testing statistical assumptions. There is also a strong shift in statistical consensus that statistical assumptions should be visually inspected. However, there are counterarguments for this. Thus, I do not agree or disagree with the authors’ comment about Mauchly’s test. Regardless, although I applaud the authors for trying different types of figures, I cannot agree with going against scientific development, and any figures on the right column are more informative than the left column figures.

Weissgerber, T. L., Winham, S. J., Heinzen, E. P., Milin-Lazovic, J. S., Garcia-Valencia, O., Bukumiric, Z., Savic, M. D., Garovic, V. D., & Milic, N. M. (2019). Reveal, Don't Conceal: Transforming Data Visualization to Improve Transparency. Circulation, 140(18), 1506–1518.

**Response 6:** We appreciate this mindful and carefully constructed comment from the reviewer. We have added a table in the results section of the paper to include the means, 95% confidence intervals, and standard deviations for each condition. We feel we have accurately represented the results of our study in the figure and table included in our manuscript.

**Reviewer #3**

**MAJOR COMMENTS - METHODS**

**Comment 1:** Pg. 8 Lines 173−174: Was a measuring tape used to obtain the exact jump distance from the distal edge of the start line (with respect to the starting position), or the value of the measurement line on the rubber mat that is closest to “the heel of the foot nearest to the start line” used to represent the estimated jump distance?

**Response 1:** We appreciate the comment from the reviewer. The standing long jump distance used in this study was measured from the heel of the foot nearest to the start line using a large rubber mat. The rubber mat had measurement lines printed on the surface. The measurement lines were in half inch increments. All measurements were converted from inches to centimeters for analysis. Measurements were taken directly from the surface of the jumping mat, a tape measure was not used.

**Comment 2:** Pg. 8 Lines 178−179: With this correction of the reported order-effect minimization method from counterbalancing to randomization, there is now a methodological concern that amongst the 29 participants, a majority of them might have performed the SLJ with one particular condition as the first of five or the last of five (so that differential effects of warmup, practice, fatigue, etc., might have influenced performance). For each of the five conditions, state the proportion of participants for which it was the first condition as well as that for which it was the fifth (last) condition.

**Response 2:** We appreciate the comment. We have added a table in the apparatus and task section that includes the count and percentage of participants that had each condition first or last.

**Comment 3:** Pg. 9 Line 199: It is unclear whether the exception refers to participants not receiving any verbal instructions before each of the two jumps in the control condition, or another unspecified provision to meet condition requirements.

**Response 3:** We appreciate this comment. We have made a change to provide more clarity that researchers provided instructions to participants before each jump. Of those provided only the internal focus instructions were used to direct attentional focus.

**Comment 4:** Pg. 10 Line 217: The p-value for the Shapiro hip dataset.

**Response 4:** The change was made.

**MAJOR COMMENTS - RESULTS**

**Comment 5:** Pg. 10 Line 217: The p-value obtained in the Shapiro-Wilk test result for your hip dataset should be 0.048.

**Response 5:** The change was made.

**Comment 6:** Pg. 10 Line 225: Consider reporting the values of standard deviation (SD) alongside these means because SD describes the sample whereas standard error (SD) is not a descriptive statistic.

**Response 6:** We have added a table in the results section of the paper to include the means, 95% confidence intervals, and standard deviations for each condition.

**MAJOR COMMENTS - DISCUSSION**

**Comment 7:** The generalizability limitation of this study should be added to the manuscript.

**Response 7:** We have added a paragraph at the end of the discussion addressing the limitations of the findings.
